# Integrating Local and Global Representation Learning for Pediatric Pneumonia Detection: A Hybrid CNN–Transformer Ensemble Framework

**DOI:** 10.3390/diagnostics16152399

**Published:** 2026-07-30

**Authors:** Ece Meltem Yalçın, Hayriye Tanyıldız, Serpil Aslan, Mustafa Yıldız, Damla Ağaçkıran, Gül Fidan

**Affiliations:** 1 Faculty of Medicine, Firat University, 23119 Elazig, Türkiye; emgurbuz@firat.edu.tr (E.M.Y.); mustafa.yildiz@firat.edu.tr (M.Y.); dr.gulfidann@gmail.com (G.F.); 2 Department of Software Engineering, Faculty of Engineering and Natural Sciences, Malatya Turgut Ozal University, 44210 Malatya, Türkiye; tanyildiz003@gmail.com; 3 Department of Pediatrics, Medical Park Bahcelievler Hospital, Altınbas University, 34180 Istanbul, Türkiye; kazardamla@gmail.com

**Keywords:** pediatric pneumonia, chest X-ray, hybrid CNN–Transformer, ensemble learning, Grad-CAM

## Abstract

**Background/Objectives**: Pneumonia remains a leading cause of childhood morbidity and mortality worldwide. Accurate interpretation of pediatric chest radiographs is challenging because of anatomical variability, subtle radiographic findings, and inter-observer variability. This study evaluates different CNN–Transformer ensemble strategies for pediatric pneumonia detection by combining complementary local and global image representations. **Methods**: Experiments were conducted on the publicly available Pediatric Pneumonia Chest X-ray dataset containing 5856 radiographs. EfficientNetV2-S was used to extract local features, whereas Swin Transformer-T modeled global anatomical relationships. Soft voting, weighted voting, and stacking were evaluated under a unified training protocol. Image preprocessing, data augmentation, and Weighted Random Sampling were applied to improve robustness and address class imbalance. Performance was assessed using an independent hold-out test set and five-fold cross-validation. Grad-CAM was used to interpret model predictions. **Results**: Ensemble learning improved classification performance compared with individual models while revealing different trade-offs among fusion strategies. The Soft Ensemble achieved the highest hold-out accuracy (96.96%) and F1-score (97.59%). The Hybrid CNN–Transformer Stacking model achieved the highest sensitivity (99.49%) and produced the fewest false-negative predictions (*n* = 2), while demonstrating the most consistent performance across five-fold cross-validation. Grad-CAM visualizations indicated that the CNN and Transformer models captured complementary radiographic information. **Conclusions**: The proposed framework demonstrates that different ensemble strategies offer distinct advantages. Soft voting provided the best overall hold-out performance, whereas stacking minimized false-negative predictions and achieved the highest sensitivity, indicating its potential for AI-assisted pediatric pneumonia screening.

## 1. Introduction

Pneumonia is a serious lower respiratory tract infection and remains one of the leading causes of morbidity and mortality among children under five years of age, particularly in low- and middle-income countries [1]. According to the World Health Organization (WHO), pneumonia accounts for a substantial proportion of childhood deaths worldwide, resulting in millions of hospital admissions and hundreds of thousands of deaths each year [2]. Although widespread immunization programs have contributed to reductions in pneumonia-related complications and mortality, the disease continues to pose a major public health challenge, especially in regions with limited access to healthcare services [3]. Early diagnosis and timely treatment are essential for improving clinical outcomes in pediatric patients, as delayed diagnosis may result in severe complications, including respiratory failure, sepsis, and long-term pulmonary sequelae [4].

Although viral infections are more common in children, bacterial pneumonia is generally associated with more severe disease manifestations and adverse clinical outcomes [4]. The disease typically presents with symptoms such as fever, cough, dyspnea, and fatigue. However, the nonspecific nature of these symptoms, particularly in younger children, may complicate the diagnostic process [5]. Consequently, radiological assessment plays a central role in the diagnosis and clinical management of pediatric pneumonia and remains one of the primary diagnostic tools in routine clinical practice [5,6,7].

Chest X-ray (CXR) remains a cornerstone of pediatric pneumonia assessment owing to its low cost, rapid availability, and widespread accessibility [5]. It enables the identification of radiographic findings associated with pneumonia, including infiltrates, consolidation, pulmonary opacities, and pleural effusion [6]. Nevertheless, the interpretation of pediatric CXR images remains challenging. Age-related variations in lung anatomy, the low contrast of infiltrative lesions, and the often subtle presentation of early-stage pneumonia can reduce diagnostic accuracy [6]. In addition, radiographic interpretation is highly dependent on clinical expertise, which may contribute to inter-observer variability and diagnostic inconsistency [6]. False-negative findings are of particular clinical concern, as they may result in missed pneumonia cases and delayed treatment, potentially leading to adverse patient outcomes [8].

Recent advances in artificial intelligence (AI) and deep learning (DL) have substantially expanded the role of automated methods in medical image analysis [9,10,11]. The increasing availability of labeled chest X-ray (CXR) datasets, together with improvements in computational resources, has facilitated the widespread adoption of deep learning techniques for medical imaging applications, including pneumonia detection [12,13]. Convolutional neural network (CNN)-based models have demonstrated strong performance in learning discriminative pathological features for pneumonia detection [9,10]. However, modeling broader anatomical context and long-range spatial dependencies remains more challenging for conventional CNN architectures than for Transformer-based models [14,15]. In contrast, Transformer-based architectures leverage self-attention mechanisms to model global contextual relationships more effectively [14,15]. Among these architectures, Swin Transformer employs a hierarchical design with shifted-window attention, enabling the extraction of both local and global image representations and demonstrating promising performance in medical imaging tasks [14,16]. EfficientNetV2, on the other hand, is an efficient CNN architecture that achieves a favorable balance between predictive performance and computational complexity through optimized training strategies and architectural design [17].

Despite the growing body of research on automated pneumonia detection, most existing studies primarily focus on either CNN-based local feature extraction or Transformer-based global representation learning [12,13,15,18]. Although hybrid CNN–Transformer architectures have attracted increasing attention, studies that systematically compare different ensemble strategies within a unified experimental framework remain relatively scarce [12,18]. In addition, the majority of previous work has emphasized overall classification performance, whereas clinically important error types, particularly false-negative predictions, have received comparatively less attention [12,13,18]. This limitation is especially relevant in pediatric pneumonia, where missed diagnoses may delay treatment and adversely affect patient outcomes [8].

To address these challenges, this study was conducted using the publicly available Pediatric Pneumonia Chest X-ray dataset, a widely adopted benchmark in the literature [19]. The class imbalance of the dataset and the considerable variability in pediatric chest radiographs present additional challenges for model development and generalization. Rather than introducing a new network architecture, the objective of this work was to systematically evaluate how different ensemble strategies influence diagnostic performance and clinically relevant error patterns, particularly false-negative predictions, by leveraging the complementary strengths of CNN- and Transformer-based representation learning. Accordingly, EfficientNetV2-S and Swin Transformer-T were integrated within a unified experimental framework, and soft voting, weighted voting, and stacking were comprehensively compared under identical training and evaluation conditions.

The main contributions of this study are summarized as follows:

- A unified CNN–Transformer framework integrating EfficientNetV2-S and Swin Transformer-T was established to exploit complementary local and global feature representations for pediatric pneumonia detection.
- Soft voting, weighted voting, and stacking ensemble strategies were systematically compared under identical training, validation, and testing protocols to provide a fair evaluation of their predictive performance and clinically relevant error patterns.
- A leakage-aware stacking framework incorporating nested cross-validation was implemented to ensure a reliable assessment of the effectiveness and generalizability of different ensemble strategies.
- Class imbalance handling strategies were comparatively evaluated through an ablation study. Based on this analysis, Weighted Random Sampling was selected as the final sampling strategy because it achieved the best overall balance between accuracy, F1-score, sensitivity, and specificity. Particular emphasis was placed on reducing clinically relevant false-negative predictions.
- Grad-CAM was employed to provide visual interpretation of model predictions by highlighting the radiographically relevant regions that contributed to the classification decisions.

## 2. Related Works

### 2.1. CNN- and Transfer Learning-Based Approaches

Deep learning has substantially improved automated pediatric pneumonia detection from chest radiographs, with early studies primarily relying on convolutional neural networks (CNNs) and transfer learning. Liang and Zheng [20] combined residual learning, dilated convolutions, and transfer learning to preserve spatial information, whereas Ayan et al. [21] demonstrated that combining multiple CNN architectures improves classification performance. Similarly, Shamrat et al. [22] proposed LungNet22, a fine-tuned VGG16-based framework that combines image preprocessing, class balancing through augmentation, and transfer learning to improve multiclass chest X-ray classification performance. Despite these advances, CNN-based models mainly capture local image patterns and have limited ability to model long-range spatial relationships [23].

Several studies have also focused on reducing computational cost while maintaining diagnostic accuracy. MobileNet-V3 [24] and LightPneumoNet [25] achieved competitive performance with considerably fewer computational resources, making them suitable for deployment in resource-constrained clinical environments. Image preprocessing has also been investigated as a complementary strategy. Mohammadi et al. [26] combined image enhancement and image fusion with a CNN-based stacking framework, whereas Shamrat et al. [27] showed that integrating CLAHE-based preprocessing, Gaussian filtering, data augmentation, and a customized MobileNetV2 architecture significantly improved multiclass lung disease classification performance. Nevertheless, most CNN-based studies have been evaluated on a single benchmark dataset under similar experimental settings. Consequently, the robustness, reproducibility, and generalizability of CNN-based approaches across diverse clinical populations remain insufficiently validated [12].

### 2.2. Transformer-Based and Hybrid CNN–Transformer Approaches

Transformer architectures have recently emerged as a prominent approach for medical image analysis, complementing conventional CNN-based models. Cahyani et al. [28] reported that Vision Transformer (ViT) achieved higher performance than several established CNN architectures for pediatric chest X-ray classification. Unlike CNNs, transformer models employ self-attention mechanisms that capture long-range dependencies and global anatomical context [14].

More recent studies have combined CNN and Transformer architectures to exploit complementary local and global feature representations [13,18,29]. These hybrid models generally outperform single-architecture networks, particularly for complex imaging tasks. However, they also introduce higher computational cost and increased optimization complexity [18,29]. In addition, most published studies emphasize overall classification performance, whereas clinically relevant aspects such as robustness, interpretability, generalization, and false-negative errors have received comparatively less attention [12,15].

### 2.3. Ensemble Learning and Explainable AI

In parallel with architectural developments, several complementary strategies have been proposed to improve model robustness, including GAN-based data augmentation, attention mechanisms, multiscale feature extraction, and activation function optimization [30,31]. These methods improve feature representation under challenging imaging conditions, although they are usually evaluated independently rather than within a unified framework [32].

Model interpretability has also received increasing attention in medical image analysis [33]. Grad-CAM has become one of the most widely used visualization techniques for identifying image regions that contribute to network predictions. For example, Akter et al. [34] combined a modified MobileNetV2 architecture with image preprocessing for COVID-19 detection from chest X-ray images, illustrating the applicability of CNN-based approaches for computer-aided chest X-ray analysis. Similarly, Lei et al. [35] combined focal loss optimization with Grad-CAM to improve both predictive performance and model interpretability. These studies indicate that explainability is becoming an important component of AI-assisted clinical decision support, complementing conventional performance metrics [33,35].

### 2.4. Research Gap and Positioning of the Proposed Study

Existing studies demonstrate the effectiveness of CNNs, Transformer architectures, preprocessing techniques, and explainability methods [12,13,15,18,30,33,35]. However, direct comparisons of multiple CNN–Transformer ensemble strategies under a unified experimental protocol remain limited. Likewise, relatively few studies examine how ensemble design influences clinically important error patterns, particularly false-negative predictions [12,13,15].

To the best of our knowledge, studies that jointly evaluate multiple CNN–Transformer ensemble strategies under identical training and evaluation settings while explicitly analyzing clinically relevant false-negative predictions remain scarce.

Table 1 summarizes representative studies according to the methodological components considered in this work, including CNN architectures, Transformer integration, hybrid representation learning, ensemble learning, explainability, and explicit false-negative analysis. The comparison shows that previous studies typically address only a subset of these components. In contrast, the proposed framework evaluates multiple CNN–Transformer ensemble strategies under identical experimental conditions while explicitly analyzing clinically relevant error patterns and model interpretability.

## 3. Materials and Methods

**Figure 1:**
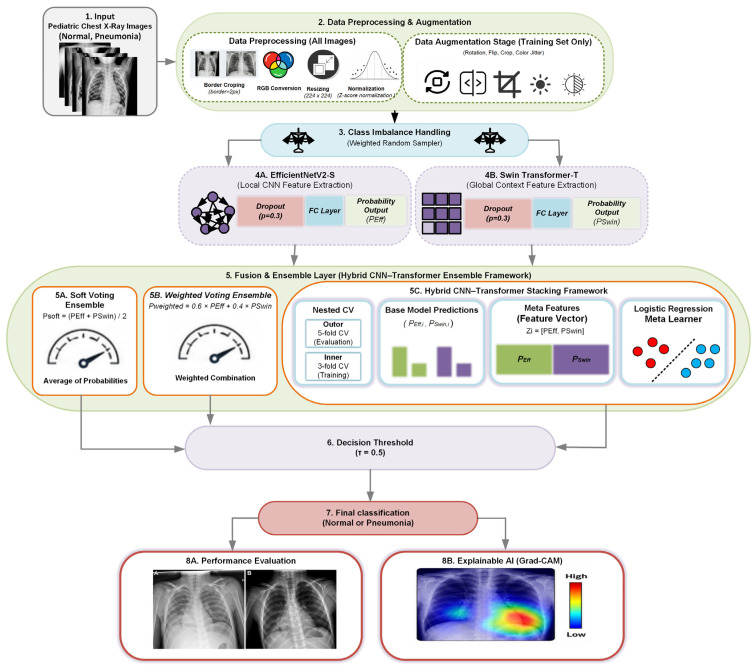
Overall architecture of the proposed Hybrid CNN–Transformer Ensemble Framework for pediatric pneumonia detection. In the performance evaluation panel (8A), (A) represents a normal chest X-ray image and (B) represents a pneumonia chest X-ray image.

This section presents in detail the dataset, preprocessing strategies, backbone networks used, ensemble learning mechanisms, and evaluation protocol of the proposed deep learning-based ensemble framework developed for the detection of pediatric pneumonia. First, the dataset and image preprocessing steps are described, followed by the introduction of basic models based on EfficientNetV2-S [17] and Swin Transformer-T [14] architectures. In the next stage, soft voting, weighted voting, and stacking-based ensemble strategies used within the Hybrid CNN–Transformer Ensemble Framework are discussed [36,37,38,39,40], and finally, the experimental protocol used to evaluate the model performance is explained. The general processing flow of the proposed method is shown in Figure 1.

### 3.1. Dataset

This study utilized the publicly available Pediatric Pneumonia Chest X-ray dataset [19], which is one of the most widely adopted benchmark datasets for automated pediatric pneumonia detection from chest radiographs. The dataset is accessible through the Kaggle platform via the Andrew MVD repository under the title “Pediatric Pneumonia Chest X-ray” and comprises anterior–posterior chest X-ray (CXR) images collected from pediatric patients. The dataset contains two diagnostic categories: NORMAL and PNEUMONIA. Owing to its relatively large sample size, clinical variability, and extensive use in previous deep learning studies, the dataset has become a reference benchmark for evaluating computer-aided pneumonia detection systems. The inclusion of radiographs acquired from pediatric patients with varying disease manifestations provides a suitable testbed for assessing the robustness and generalization capability of deep learning models. The dataset consists of 5856 original chest X-ray images. No additional images were generated or permanently added to the dataset.

Prior to model development, a Python-based image integrity analysis was performed to assess potential data leakage across the official training and test partitions, as well as the validation folds generated during cross-validation. Exact duplicate images were identified using content-based image hashing, while near-duplicate images were screened using perceptual hashing. No exact or near-duplicate images were identified across these data partitions, confirming that the same or visually equivalent radiographs were not shared between the official training and test partitions or the validation folds generated during cross-validation.

A notable characteristic of the dataset is its class imbalance, with the pneumonia category containing substantially more samples than the normal category. Such imbalance may bias the optimization process toward the majority class, resulting in inflated overall accuracy while masking clinically important classification errors. In particular, an increased tendency toward false-negative predictions may reduce the clinical utility of automated screening systems, as undetected pneumonia cases can delay diagnosis and treatment. To mitigate this issue and promote more balanced decision boundaries during model training, class-balancing strategies were incorporated into the experimental workflow. The objective was not only to improve overall classification performance but also to reduce the risk of clinically critical false-negative outcomes.

Table 2 summarizes the composition of the Pediatric Pneumonia Chest X-ray dataset used throughout this study.

### 3.2. Image Preprocessing and Data Augmentation

Pediatric chest radiographs are inherently heterogeneous due to variations in acquisition devices, imaging protocols, patient positioning, and anatomical characteristics across different age groups. Such variability may adversely affect model generalization and increase susceptibility to overfitting. To mitigate these challenges, a standardized image preprocessing pipeline and an online data augmentation strategy were implemented prior to model training.

- Image Preprocessing: The original chest radiographs were acquired in grayscale format with varying spatial resolutions. To ensure compatibility with the input specifications of both EfficientNetV2-S and Swin Transformer-T architectures, all images were converted to three-channel RGB representations. Although the diagnostic information remained unchanged, this conversion enabled direct utilization of ImageNet-pretrained model weights and ensured architectural consistency during transfer learning. Subsequently, border cropping was performed to remove non-diagnostic regions, including black margins, scanning artifacts, and acquisition-related background areas that do not contribute to clinical interpretation. Eliminating such irrelevant regions reduced the influence of noise and allowed the models to focus more effectively on anatomically meaningful structures. Following artifact removal, all images were resized to a fixed resolution of 224 × 224 pixels, corresponding to the native input size required by both backbone architectures. Standardizing image dimensions ensured computational efficiency and provided a consistent spatial representation across all samples. Finally, pixel intensity values were normalized prior to model ingestion. This normalization step reduced intensity-scale variability among radiographs, facilitated numerical stability during optimization, and promoted faster convergence of the gradient-based learning process.
- Data Augmentation: To enhance model generalization and reduce overfitting, online data augmentation was applied exclusively to the training set during model training. Accordingly, the dataset size remained unchanged, with augmented images generated on-the-fly during each training epoch rather than being permanently stored. The augmentation strategy was designed to simulate realistic variations commonly encountered in clinical radiographic practice while preserving diagnostically relevant pulmonary structures.

The following augmentation operations were employed:

o  Random rotation within ±15°;
o  Horizontal flipping;
o  Random resized cropping with a scaling range of 0.8–1.0;
o  Random brightness adjustment and random contrast adjustment.

Moderate rotational transformations were introduced to account for slight patient-positioning inconsistencies during image acquisition. Random resized cropping encouraged the models to learn robust representations from different spatial regions while maintaining the overall anatomical context. Brightness and contrast perturbations simulated variations originating from imaging devices, acquisition settings, and post-processing protocols. By exposing the models to a broader range of clinically plausible image variations during training, the augmentation pipeline improved robustness against acquisition-related variability and enhanced the generalization capability of the proposed framework on previously unseen radiographs.

- Handling Class Imbalance: To alleviate the bias toward the majority class caused by the imbalanced class distribution, Weighted Random Sampling [37] was employed. In this method, each sample is assigned a weight inversely proportional to the frequency of the class to which it belongs. Thus, it is aimed that both classes contribute to the optimization process in a more balanced manner during training. This approach provides a significant advantage, especially in terms of reducing false negative results.

To justify the choice of the imbalance handling strategy, an ablation study was conducted by comparing Weighted Random Sampling with four commonly used alternatives: no balancing, class-weighted BCE loss, focal loss, and balanced batch sampling. As summarized in Table 3, Weighted Random Sampling achieved the highest overall classification performance, yielding the best accuracy (0.9696), F1-score (0.9759), sensitivity (0.9872), and specificity (0.9402). Based on these results, Weighted Random Sampling was adopted for all subsequent experiments reported in this study.

### 3.3. Base Deep Learning Architectures

In this study, two different deep learning backbone networks were employed for pediatric pneumonia classification: EfficientNetV2-S and Swin Transformer-Tiny (Swin-T). These architectures were selected because they provide complementary capabilities in local and global representation learning. EfficientNetV2-S demonstrates strong performance in learning local radiographic patterns, whereas Swin Transformer-T provides an advantage in modeling long-range spatial dependencies.

#### 3.3.1. EfficientNetV2-S

**Figure 2:**
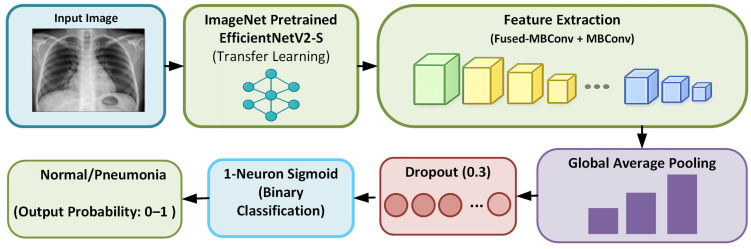
EfficientNetV2-S architecture used in this study.

EfficientNetV2-S is a CNN-based architecture developed to achieve a balanced trade-off between model accuracy and computational efficiency [17]. Compared with previous EfficientNet variants, it exhibits faster convergence and more stable training behavior owing to its combination of mobile inverted bottleneck convolution layers and progressive learning strategies. The architecture of the EfficientNetV2-S model used in this study is illustrated in Figure 2. In this study, the EfficientNetV2-S model, pre-trained on ImageNet, was utilized within a transfer learning framework. The original classification layer was removed and replaced with a dropout layer (*p* = 0.3) and a sigmoid-activated binary classification head consisting of a single output neuron. This modification enabled the learned feature representations of the model to be adapted to the pediatric pneumonia detection task.

#### 3.3.2. Swin Transformer-Tiny

**Figure 3:**
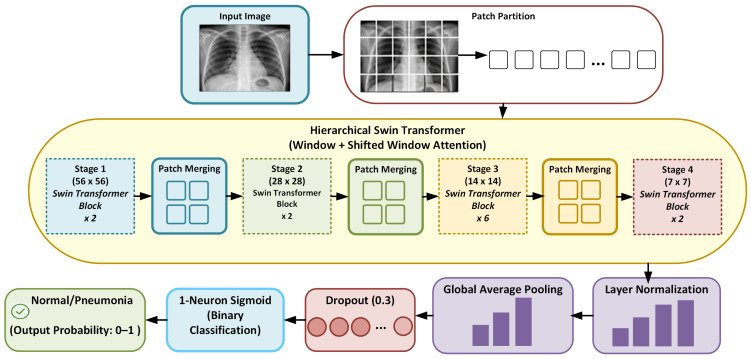
Swin Transformer-T architecture used in this study.

Swin Transformer-Tiny (Swin-T) is a hierarchical transformer-based vision architecture that employs a self-attention mechanism within non-overlapping local windows and facilitates information exchange between windows through a shifted-window strategy [14]. This design enables the learning of both local and global image representations while reducing computational complexity. The architecture of the Swin Transformer-T model used in this study is presented in Figure 3. In the context of thoracic radiographs, Swin-T offers a significant advantage by modeling not only localized lesion regions but also infiltrative spread patterns and broader anatomical relationships [14,15]. In this study, an ImageNet pre-trained Swin-T model was utilized. The original classification head was removed and replaced with a dropout layer (*p* = 0.3) and a sigmoid-activated binary classification layer.

The implementation settings used during model training are summarized in Table 4. Unless otherwise specified, the same training configuration was applied to all individual and ensemble models throughout the experiments.

### 3.4. Ensemble Learning Framework

Studies on pediatric pneumonia detection have shown that CNN-based architectures provide distinct advantages in learning local radiographic patterns, whereas Transformer-based models are more effective in modeling broader anatomical contexts and long-range spatial relationships. Nevertheless, even high-performing individual models may exhibit different error profiles depending on the data distribution and may generate inconsistent predictions for certain sample groups. This issue is particularly relevant in pediatric pneumonia diagnosis, where false-negative predictions may lead to clinically significant consequences. Consequently, ensemble learning has emerged as a widely adopted strategy for combining models with complementary representation-learning capabilities to develop more balanced, stable, and reliable classification systems [38].

In this study, EfficientNetV2-S, which demonstrated strong local feature extraction capability, and Swin Transformer-T, which effectively captures global contextual relationships, were integrated within a Hybrid CNN–Transformer Ensemble Framework. The objective was to enrich the representation space by combining the fine-grained tissue and opacity representations learned by EfficientNetV2-S with the global anatomical context information captured by Swin Transformer-T. By leveraging the complementary strengths of CNN- and Transformer-based learning mechanisms, the framework aimed to achieve more reliable generalization performance across different patient samples and data partitions.

To investigate the effectiveness of decision-level fusion, three ensemble strategies were evaluated [38]:

(1) Soft Voting;
(2) Weighted Voting;
(3) Hybrid CNN–Transformer Stacking Approach.

All ensemble methods were assessed using the same dataset, training protocol, and evaluation criteria to ensure a fair comparison. The classification performance, generalizability, and impact of each ensemble strategy on false-negative predictions were subsequently analyzed.

#### 3.4.1. Soft Voting

Soft voting is one of the most widely used decision-level fusion strategies in ensemble learning. In this approach, the class probabilities generated by the base models are combined with equal weights, and the final decision is determined based on the aggregated probability value. Consequently, each model contributes equally to the decision-making process. In this study, the posterior probabilities produced by the base models were combined with equal contributions. The final probability value was calculated as follows:

(1) $$p_{soft}(x)=\frac{p_{Eff}(x)+p_{Swin}(x)}{2}$$

Here, $p_{Eff}\left(x\right)$ represents the pneumonia probability predicted by the EfficientNetV2-S model, and $p_{Swin}(x)$ represents the pneumonia probability predicted by the Swin Transformer-T model.

The soft voting strategy can contribute to more stable classification outcomes by balancing variations in the probability outputs generated by different architectures.

#### 3.4.2. Weighted Voting

While the soft voting approach assigns equal contributions to all models, the weighted voting strategy allows different weights to be assigned according to the performance characteristics of individual models [36]. This approach enables a model demonstrating superior performance on specific metrics to exert greater influence on the final decision. In this study, the contributions of the base models were not considered equal. Based on preliminary experiments conducted on the validation data, the EfficientNetV2-S model was assigned a higher weight because it demonstrated stronger performance in terms of positive-class sensitivity than the Swin Transformer-T model. Accordingly, a weight combination of 0.6 for EfficientNetV2-S and 0.4 for Swin Transformer-T was adopted for the weighted voting strategy. The final probability value was calculated as follows:

(2) $$p_{weighted}(x)=0.6\times p_{Eff}(x)+0.4\times p_{Swin}(x)$$

The resulting probability value was subsequently converted into a binary class label using a decision threshold of 0.5. The resulting probability value was subsequently converted into a binary class label using a decision threshold of 0.5. A common decision threshold of 0.5 was applied to all individual and ensemble models to ensure a fair and consistent comparison under identical experimental conditions. The aim of this study was to compare the relative performance of different ensemble strategies rather than to optimize the operating threshold for a specific clinical application. Therefore, all reported performance metrics were obtained using the same decision threshold.

#### 3.4.3. Hybrid CNN–Transformer Stacking Framework

Stacking is a hierarchical ensemble learning approach in which the outputs generated by multiple models are combined through a second-level learner to produce the final classification decision. Unlike voting-based methods, stacking can learn complementary relationships among base models and provides a more flexible decision-making mechanism. In this study, a stacking-based ensemble strategy was employed to combine the outputs of the EfficientNetV2-S and Swin Transformer-T models more effectively. The meta-learner was trained using the probability outputs generated by the base models to produce the final classification. To obtain an unbiased estimate of performance during meta-learner training, a nested cross-validation-supported training procedure was adopted. Within this framework, the meta-learner was trained and evaluated exclusively using out-of-fold (OOF) predictions. The meta-feature vector constructed for each sample was defined as follows:

(3) $$z_{i}=\left[p_{Eff,i},\;p_{Swin,i}\right]$$

Here, $p_{Eff,i}$ and $p_{Swin,i}$ represent the pneumonia probabilities generated by the EfficientNetV2-S and Swin Transformer-T models for the (*i*)-th sample, respectively.

**Figure 4:**
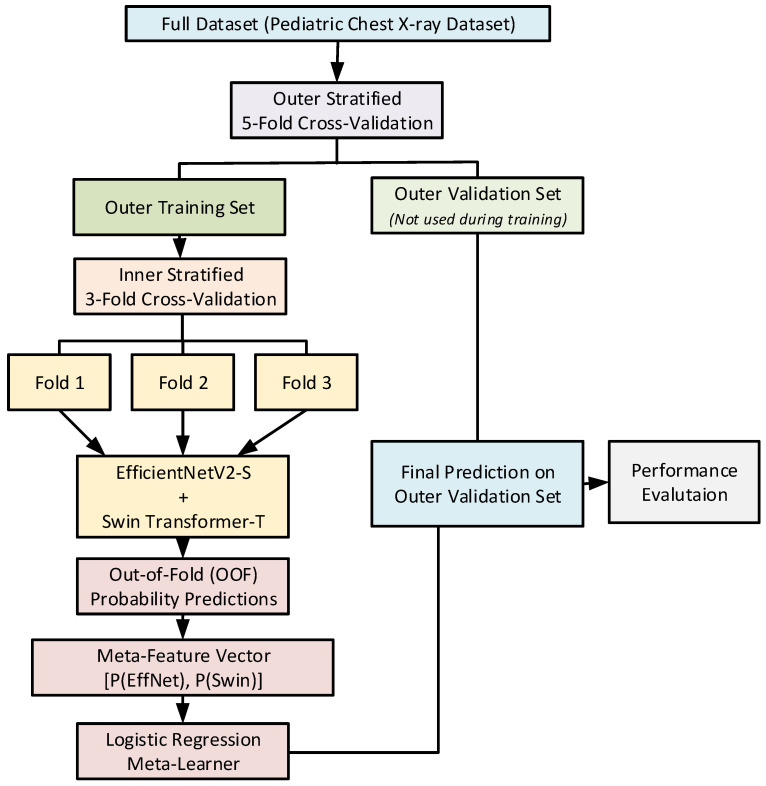
Workflow of the leakage-free nested cross-validation stacking framework.

To ensure a leakage-free stacking procedure, a nested cross-validation strategy was adopted (Figure 4). The entire dataset was first divided using an outer stratified five-fold cross-validation, where one fold was reserved as the outer validation set, and the remaining four folds were used for training. Within each outer training set, an inner stratified three-fold cross-validation was performed to train the EfficientNetV2-S and Swin Transformer-T base models. During each inner fold, the trained models generated probability predictions for the corresponding validation fold, producing out-of-fold (OOF) predictions for all samples in the outer training set. These predictions were combined to form the meta-feature vectors used to train the Logistic Regression meta-learner. The trained base models were then applied to the independent outer validation fold, and their probability predictions were combined by the Logistic Regression meta-learner to obtain the final classification. The same procedure was repeated for each outer fold. Since the outer validation set was never used during base-model or meta-learner training, information leakage between training and evaluation was avoided.

Logistic Regression [40] was selected as the meta-learner due to its low computational complexity, strong probability calibration capability, and interpretable decision mechanism. Compared with more complex meta-learning models, Logistic Regression can provide a more stable and transparent decision boundary while reducing the risk of overfitting in limited-scale medical imaging datasets.

The proposed Hybrid CNN–Transformer Stacking Framework integrates CNN-based local representation learning and Transformer-based global representation learning at the meta-learning level. By leveraging the complementary feature representations and distinct error profiles of the two architectures, the framework seeks to generate more reliable classification decisions. The primary objective of this approach is not only to improve overall predictive performance but also to establish a more stable, generalizable, and clinically reliable decision-support mechanism for pediatric pneumonia detection.

### 3.5. Evaluation Protocol

A two-stage evaluation protocol was implemented to comprehensively assess the performance of the proposed Hybrid CNN–Transformer Ensemble Framework: (i) independent hold-out test evaluation and (ii) five-fold cross-validation analysis [41,42]. This evaluation strategy enabled the assessment of both the model’s generalization capability on previously unseen samples and the stability of its performance across different data partitions.

- Hold-Out Test Evaluation: In the first stage, experiments were conducted using an independent hold-out test set established according to the official training–test split of the dataset. The official training partition was used for model development, whereas the official test partition was reserved exclusively for the final evaluation. For the proposed stacking framework, all model development was performed using only the training partition, following the leakage-free nested cross-validation procedure described in Section 3.4.3. This evaluation provides a realistic assessment of model performance by measuring predictive capability on images that were not encountered during training. In medical imaging applications, independent test evaluation serves as an important benchmark for assessing a model’s ability to generalize to new and unseen samples.
- Five-Fold Cross-Validation: In the second stage, five-fold cross-validation was performed to reduce performance variability associated with a particular data partition. The dataset was divided into five subsets, and in each iteration, four folds were used for training while the remaining fold was used for validation. This procedure was repeated until each fold had served as the validation set once, after which the average performance across all folds was reported. The proposed Hybrid CNN–Transformer Stacking Framework additionally incorporated a nested cross-validation strategy. Within this procedure, the meta-learner was trained exclusively on out-of-fold (OOF) predictions. Meta-feature vectors constructed from the OOF predictions generated by the EfficientNetV2-S and Swin Transformer-T models were provided as inputs to the Logistic Regression-based meta-learner. This design ensured that the meta-learner was trained using independent predictions during the stacking process, thereby facilitating a more reliable and unbiased estimation of model performance.

### 3.6. Computational Complexity Analysis

To assess the computational efficiency and deployment feasibility of the evaluated models, the number of trainable parameters, MACs, FLOPs, training time, inference time per image, and throughput were measured under the same experimental settings. All experiments were conducted using the PyTorch 2.11.0 framework on an NVIDIA Tesla T4 GPU (NVIDIA Corporation, Santa Clara, CA, USA). The results are summarized in Table 5.

## 4. Results

This section presents the experimental results of the proposed Hybrid CNN–Transformer Ensemble Framework. Model performance was evaluated using Accuracy (ACC), Area Under the Curve (AUC), Average Precision (AP), F1-score, Sensitivity, and Specificity metrics. In addition, confusion matrix analyses were conducted to examine error distributions in greater detail, and Grad-CAM-based visual interpretability analyses were performed to assess the transparency of model decisions.

### 4.1. Hold-Out Test Results

The hold-out test results presented in Table 6 and Table 7 indicate that both individual and ensemble models achieved high classification performance, although notable differences were observed in their error distributions and the trade-off between sensitivity and specificity. The Soft Ensemble approach achieved the best overall balance between sensitivity and specificity, with an accuracy of 0.9696, an F1-score of 0.9759, an AUC of 0.9921, and an AP of 0.9936, demonstrating the effectiveness of probability-level fusion between CNN- and Transformer-based models.

Among the individual models, EfficientNetV2-S provided the most balanced performance, achieving high sensitivity (0.9897) while maintaining relatively high specificity (0.9188). ResNet50 reduced the number of false-negative predictions to three, whereas DenseNet121 and MobileNetV3 detected all pneumonia cases without false negatives. However, this improvement was accompanied by a substantial increase in false-positive predictions, resulting in noticeably lower specificity and overall accuracy. Although Swin Transformer-T achieved the highest specificity among the individual models, it produced more false negatives than the CNN-based architectures. These results indicate that different transfer learning architectures exhibit distinct diagnostic characteristics rather than consistently superior performance.

Among the ensemble methods, the Hybrid CNN–Transformer Stacking model achieved the highest sensitivity (0.9949) and produced only two false-negative predictions, indicating its effectiveness in minimizing missed pneumonia cases. In contrast, the Soft Ensemble provided the best overall balance between sensitivity and specificity, while the Weighted Ensemble showed intermediate performance across the evaluated metrics.

Overall, the hold-out evaluation suggests that the ensemble strategies provide a more balanced trade-off between false-positive and false-negative predictions than the individual transfer learning models, supporting their potential application in computer-aided pediatric pneumonia screening.

**Figure 5:**
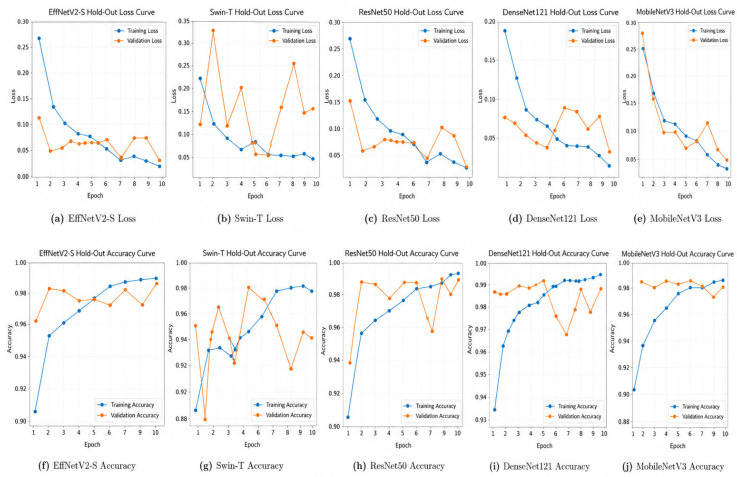
Training and validation loss and accuracy curves of the representative transfer learning models under the hold-out evaluation.

The training and validation learning curves of the representative transfer learning models are shown in Figure 5. Overall, the models exhibited stable convergence throughout training, with training and validation curves following similar trends. Although minor fluctuations were observed, particularly for Swin Transformer-T, no persistent divergence between the training and validation curves was evident. These results suggest that the evaluated models did not exhibit substantial overfitting under the hold-out evaluation protocol.

To assess the robustness of the reported results, approximate 95% confidence intervals were calculated for all evaluation metrics (Table 7). Pairwise statistical comparisons were also performed using McNemar’s test and DeLong’s test (Table 8). Although the confidence intervals of the best-performing models largely overlapped and no statistically significant differences were observed in the hold-out evaluation (Holm-adjusted *p* > 0.05 for all comparisons), the confusion matrices revealed differences in error distribution. In particular, the Hybrid CNN–Transformer Stacking model produced the fewest false-negative predictions, whereas the Soft Ensemble achieved the best overall performance on the hold-out test.

### 4.2. Five-Fold Cross-Validation Results

To assess model performance under different data partitions, the experiments were repeated using a five-fold cross-validation protocol. The results presented in Table 9 and Table 10 demonstrate consistently high performance across all evaluated models while revealing notable differences in their error distributions and class-specific performance.

Among the individual models, DenseNet121 achieved the highest accuracy (0.9764 ± 0.004) and F1-score (0.9838 ± 0.003), followed by MobileNetV3 and EfficientNetV2-S. Although DenseNet121 and MobileNetV3 exhibited strong overall performance, EfficientNetV2-S maintained a more balanced trade-off between sensitivity and specificity. Swin Transformer-T achieved the highest specificity (0.9766 ± 0.005) but produced considerably more false-negative predictions (251) than the CNN-based architectures. ResNet50 provided competitive performance but did not outperform the leading CNN models across the evaluated metrics.

Among the ensemble methods, the Hybrid CNN–Transformer Stacking approach achieved the highest sensitivity (0.9796 ± 0.004) and produced the fewest false-negative predictions (87). In contrast, the Soft Ensemble achieved the highest AUC (0.9968 ± 0.001) and AP (0.9988 ± 0.000), while the Weighted Ensemble consistently yielded intermediate performance across the evaluated metrics.

The aggregated confusion matrices in Table 9 further illustrate the differences in prediction behavior among the evaluated models. While the Hybrid CNN–Transformer Stacking approach minimized false-negative predictions, the Soft Ensemble produced fewer false positives than the other ensemble methods. These findings highlight the complementary characteristics of the proposed ensemble strategies, which achieved competitive performance while providing different trade-offs between sensitivity and specificity compared with the individual transfer learning models under repeated data partitioning.

**Figure 6:**
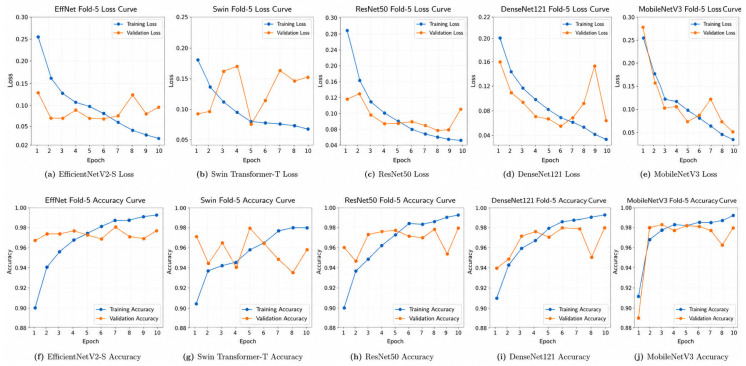
Training and validation loss and accuracy curves of the representative transfer learning models under the five-fold cross-validation evaluation.

The training and validation learning curves further support these findings. As shown in Figure 6, the training and validation loss decreased consistently throughout the training process, while the corresponding accuracy curves remained closely aligned across epochs. Although minor fluctuations were observed, particularly for Swin Transformer-T and DenseNet121, no persistent divergence between the training and validation curves was evident, indicating stable convergence and no substantial overfitting during five-fold cross-validation.

To determine whether the observed performance differences were statistically significant, pairwise comparisons were performed using McNemar’s test for classification outcomes and DeLong’s test for ROC-AUC values (Table 11). Although the improvement of the Hybrid CNN–Transformer Stacking model over EfficientNetV2-S was modest (0.22% in accuracy), McNemar’s test indicated a statistically significant difference in classification outcomes (Holm-adjusted *p* = 0.000054). In contrast, DeLong’s test found no statistically significant difference in ROC-AUC between the two models (Holm-adjusted *p* = 1.0000), suggesting that the observed improvement was related to classification decisions rather than overall ranking performance.

### 4.3. Grad-CAM-Based Visual Explainability Analysis

**Figure 7:**
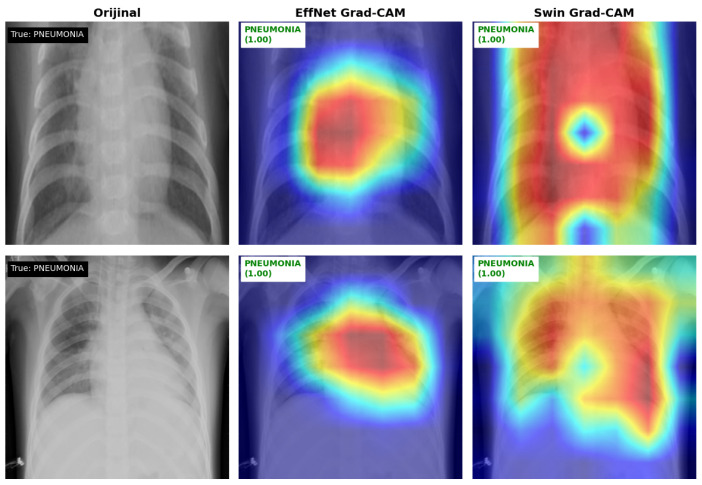
Grad-CAM visualizations of representative pediatric pneumonia chest X-ray images generated by EfficientNetV2-S and Swin Transformer-T. In the Grad-CAM visualizations, warmer colors indicate higher activation, whereas cooler colors indicate lower activation.

To improve the interpretability of the proposed framework, Grad-CAM visualizations were generated for the EfficientNetV2-S and Swin Transformer-T models. Representative pediatric chest X-ray images and their corresponding activation maps are presented in Figure 7.

As shown in Figure 3, the EfficientNetV2-S model generated compact activation regions that were primarily concentrated around radiographic findings associated with pulmonary infiltration and opacity. These activation patterns indicate that the CNN-based model relied mainly on localized image features when identifying pneumonia cases. In contrast, the Swin Transformer-T model produced activation maps distributed over broader anatomical regions of the thorax. Rather than focusing on a single localized area, the model incorporated a wider spatial context into its predictions, reflecting the global representation learning capability of Transformer-based architectures.

The Grad-CAM visualizations therefore revealed different activation patterns for the two backbone models. EfficientNetV2-S focused primarily on localized pulmonary abnormalities, whereas Swin Transformer-T attended to broader anatomical regions.

## 5. Discussion

The proposed framework was evaluated using both an independent hold-out test and five-fold cross-validation. The results show that each ensemble strategy has different strengths for pediatric pneumonia detection. The Soft Ensemble achieved the best overall performance on the hold-out test set, whereas the Hybrid CNN–Transformer Stacking model consistently produced the fewest false-negative predictions during cross-validation. These findings suggest that the choice of an ensemble strategy should depend on the intended clinical application rather than overall accuracy alone.

Table 12 compares the proposed framework with representative studies on pediatric pneumonia detection. Previous studies mainly focused on CNN-based transfer learning or Vision Transformer models and were generally evaluated using a single model or a single experimental protocol. In contrast, this study compares multiple CNN–Transformer ensemble strategies under the same experimental conditions using both hold-out testing and five-fold cross-validation.

Additional experiments with ResNet50, DenseNet121, and MobileNetV3 showed that different CNN backbones produce different trade-offs between sensitivity and specificity. DenseNet121 and MobileNetV3 achieved perfect sensitivity on the hold-out test set but generated substantially more false-positive predictions. Overall, combining complementary CNN and Transformer representations resulted in competitive performance while maintaining a balanced trade-off between sensitivity and specificity.

A strength of this study is the systematic evaluation of different ensemble strategies under identical experimental conditions. In addition to overall classification performance, the analysis also considered clinically important error patterns, particularly false-negative predictions.

From a clinical perspective, reducing false-negative predictions is important because missed pneumonia cases may delay diagnosis and treatment. Although the improvements in the overall performance metrics were modest, the stacking strategy consistently reduced false-negative predictions, whereas the Soft Ensemble provided a better balance between sensitivity and specificity. These findings indicate that different ensemble strategies may be preferred depending on the clinical objective.

This study has several limitations. The experiments were conducted using a single publicly available pediatric chest X-ray dataset without external validation. Grad-CAM was used only for qualitative interpretation and was not evaluated by expert radiologists. In addition, the proposed framework was evaluated only for binary classification. Future studies should include external multicenter validation, quantitative localization analysis, expert radiologist assessment of Grad-CAM results, and multimodal clinical information.

## 6. Conclusions

This study presented a Hybrid CNN–Transformer ensemble framework for pediatric pneumonia detection by integrating EfficientNetV2-S and Swin Transformer-T. Soft voting, weighted voting, and stacking were evaluated under the same experimental protocol.

The results showed that the Soft Ensemble achieved the best overall performance on the independent hold-out test set, whereas the Hybrid CNN–Transformer Stacking model consistently produced the fewest false-negative predictions and the highest sensitivity during five-fold cross-validation. These findings indicate that different ensemble strategies may be preferred depending on the intended clinical application, particularly in screening settings where minimizing missed pneumonia cases is a priority.

The proposed framework achieved competitive performance by combining the complementary strengths of CNN and Transformer models. Further validation using larger multicenter datasets will be necessary to confirm its generalizability and clinical applicability.

## Figures and Tables

**Table 1:** Comparison of representative pediatric pneumonia detection studies with respect to key methodological components and the positioning of the proposed framework.

Reference	CNN	Transformer	Hybrid	Ensemble	Explainability	False-Negative Error Analysis
Liang & Zheng [20]	✓	–	–	–	–	–
Ayan et al. [21]	✓	–	–	✓	–	–
Shamrat et al. [22]	✓	–	–	–	✓	–
MobileNet-V3 [24]	✓	–	–	–	–	–
LightPneumoNet [25]	✓	–	–	–	–	–
Mohammadi et al. [26]	✓	–	–	✓	–	–
Shamrat et al. [27]	✓	–	–	–	✓	–
Cahyani et al. [28]	✓	✓	–	–	–	–
Mustapha et al. [18]	✓	✓	✓	–	–	–
Akter et al. [34]	✓	–	–	–	–	–
Lei et al. [35]	✓	–	–	–	✓	–
Proposed Framework	✓	✓	✓	✓	✓	✓

Note: ✓ = present; – = not present.

**Table 2:** Summary statistics of the Pediatric Pneumonia Chest X-ray dataset.

Dataset Attribute	Description
Dataset name	Pediatric Pneumonia Chest X-ray
Source	Kaggle (andrewmvd)
Total images	5856
Normal images	1583
Pneumonia images	4273
Image format	JPEG
Age group	Pediatric (especially 1–5 years)
Classes	NORMAL, PNEUMONIA
Splits	Official train/test split (validation folds generated during stratified 5-fold cross-validation)
Standard view	Anterior–Posterior CXR
Expert verified labels	Yes

**Table 3:** Ablation study of class imbalance handling strategies evaluated on the hold-out test set.

Imbalance Handling Strategy	Accuracy	F1-Score	Sensitivity	Specificity
No Balancing	0.9584	0.9682	0.9564	0.9145
Class-Weighted BCE Loss	0.9621	0.9716	0.9744	0.9017
Focal Loss	0.9668	0.9751	0.9821	0.9188
Balanced Batch Sampling	0.9647	0.9738	0.9795	0.9103
Weighted Random Sampling (Proposed)	0.9696	0.9759	0.9872	0.9402

**Table 4:** Implementation details used for training the proposed framework.

Parameter	Value
Framework	PyTorch 2.11.0 (CUDA 12.8)
Python	3.12.13
Optimizer	AdamW
Learning rate	5 × 10^−5^
Batch size	16
Epochs	10
Loss function	BCEWithLogitsLoss
Learning rate scheduler	CosineAnnealingLR
Early stopping	Not applied
Random seed	42
Transfer learning	ImageNet-pretrained weights
Fine-tuning	Full fine-tuning (all layers)

**Table 5:** Computational complexity and runtime comparison of the evaluated models.

Model	Params (M)	MACs (G)	FLOPs (G)	Train Time (s/epoch)	Total Time (s)	Infer. Time (ms)	Throughput (img/s)
EfficientNetV2-S	20.18	2.901	5.801	239.43	2394.32	12.25	81.65
Swin Transformer-T	27.52	2.977	5.955	156.10	1561.04	12.77	78.33

Abbreviations: MACs, multiply–accumulate operations; FLOPs, floating-point operations.

**Table 6:** Hold-out test confusion matrices of individual and ensemble models for pediatric pneumonia detection.

Model	TN	FP	FN ↓	TP
EfficientNetV2-S	215	19	4	386
Swin Transformer-T	217	17	8	382
ResNet50	191	43	3	387
DenseNet121	173	61	0	390
MobileNetV3	168	66	0	390
Soft Ensemble	220	14	5	385
Weighted Ensemble	216	18	5	385
Hybrid CNN–Transformer Stacking	207	27	2	388

Note: ↓ indicates that lower values represent better performance.

**Table 7:** Performance metrics of individual and ensemble models evaluated on the hold-out test set (with approximate 95% confidence intervals).

Method	ACC	AUC	AP	F1	Sensitivity	Specificity
EfficientNetV2-S	0.9631 (0.9470–0.9788)	0.9907 (0.9820–0.9970)	0.9926 (0.9860–0.9984)	0.9711 (0.9570–0.9824)	0.9897 (0.9818–0.9978)	0.9188 (0.8790–0.9510)
Swin Transformer-T	0.9599 (0.9440–0.9755)	0.9918 (0.9830–0.9973)	0.9944 (0.9882–0.9988)	0.9683 (0.9542–0.9804)	0.9795 (0.9690–0.9920)	0.9274 (0.8900–0.9580)
ResNet50	0.9263 (0.9050–0.9460)	0.9831 (0.9720–0.9920)	0.9862 (0.9780–0.9930)	0.9440 (0.9250–0.9610)	0.9923 (0.9850–0.9990)	0.8174 (0.7680–0.8640)
DenseNet121	0.9022 (0.8790–0.9240)	0.9750 (0.9630–0.9860)	0.9812 (0.9710–0.9900)	0.9275 (0.9080–0.9460)	1.0000 (0.9910–1.0000)	0.7393 (0.6840–0.7920)
MobileNetV3	0.8942 (0.8700–0.9170)	0.9720 (0.9590–0.9840)	0.9780 (0.9670–0.9880)	0.9219 (0.9010–0.9410)	1.0000 (0.9910–1.0000)	0.7179 (0.6620–0.7720)
Soft Ensemble	0.9696 (0.9540–0.9838)	0.9921 (0.9840–0.9975)	0.9936 (0.9872–0.9985)	0.9759 (0.9630–0.9850)	0.9872 (0.9800–0.9960)	0.9402 (0.9040–0.9680)
Weighted Ensemble	0.9631 (0.9470–0.9788)	0.9920 (0.9840–0.9973)	0.9935 (0.9870–0.9984)	0.9710 (0.9570–0.9820)	0.9872 (0.9800–0.9960)	0.9231 (0.8860–0.9540)
Hybrid CNN–Transformer Stacking	0.9535 (0.9370–0.9690)	0.9921 (0.9842–0.9976)	0.9937 (0.9873–0.9986)	0.9640 (0.9500–0.9760)	0.9949 (0.9870–1.0000)	0.8846 (0.8440–0.9220)

**Table 8:** Pairwise statistical comparison of the hold-out test results using McNemar’s and DeLong’s tests.

Comparison	Discordant Pairs	Holm-Adjusted *p* (McNemar)	Significant	ΔAUC	Holm-Adjusted *p* (DeLong)	Significant
Swin Transformer-T vs. EfficientNetV2-S	18	1.0000	No	−0.002487	1.0000	No
Soft Ensemble vs. EfficientNetV2-S	10	1.0000	No	−0.000186	1.0000	No
Weighted Ensemble vs. EfficientNetV2-S	9	1.0000	No	−0.000329	1.0000	No
Hybrid CNN–Transformer Stacking vs. EfficientNetV2-S	9	1.0000	No	−0.000099	1.0000	No

Abbreviations: ΔAUC, difference in the area under the receiver operating characteristic curve. Holm-adjusted *p*-values were calculated using the Holm correction for multiple pairwise comparisons.

**Table 9:** Aggregated confusion matrix results obtained from five-fold cross-validation.

Model	TN	FP	FN	TP
EfficientNetV2-S	1520	63	104	4169
Swin-T	1546	37	251	4022
ResNet50	1539	44	160	4113
DenseNet121	1542	41	97	4176
MobileNetV3	1521	62	98	4175
Soft Ensemble	1538	45	134	4139
Weighted Ensemble	1528	55	116	4157
Hybrid CNN–Transformer Stacking	1516	67	87	4186

**Table 10:** Comparative performance of individual and ensemble models under five-fold cross-validation (mean ± SD).

Method	ACC	AUC	AP	F1	Sensitivity	Specificity
EfficientNetV2-S	0.9715 ± 0.005	0.9955 ± 0.001	0.9982 ± 0.000	0.9803 ± 0.004	0.9757 ± 0.006	0.9602 ± 0.008
Swin Transformer-T	0.9508 ± 0.009	0.9964 ± 0.001	0.9987 ± 0.000	0.9650 ± 0.006	0.9413 ± 0.010	0.9766 ± 0.005
ResNet50	0.9652 ± 0.006	0.9959 ± 0.001	0.9984 ± 0.000	0.9758 ± 0.004	0.9626 ± 0.006	0.9722 ± 0.006
DenseNet121	0.9764 ± 0.004	0.9966 ± 0.001	0.9988 ± 0.000	0.9838 ± 0.003	0.9773 ± 0.005	0.9741 ± 0.005
MobileNetV3	0.9727 ± 0.005	0.9962 ± 0.001	0.9986 ± 0.000	0.9812 ± 0.004	0.9771 ± 0.005	0.9608 ± 0.007
Soft Ensemble	0.9694 ± 0.005	0.9968 ± 0.001	0.9988 ± 0.000	0.9788 ± 0.004	0.9686 ± 0.006	0.9716 ± 0.006
Weighted Ensemble	0.9708 ± 0.004	0.9967 ± 0.001	0.9987 ± 0.000	0.9798 ± 0.004	0.9729 ± 0.005	0.9653 ± 0.007
Hybrid CNN–Transformer Stacking	0.9737 ± 0.004	0.9967 ± 0.001	0.9987 ± 0.000	0.9819 ± 0.003	0.9796 ± 0.004	0.9577 ± 0.008

**Table 11:** Pairwise statistical comparison of five-fold cross-validation results using McNemar’s and DeLong’s tests.

Comparison	Holm-Adjusted *p* (McNemar)	Significant	ΔAUC	Holm-Adjusted *p* (DeLong)	Significant
Hybrid CNN–Transformer Stacking vs. EfficientNetV2-S	0.000054	Yes	−0.00024	1.00000	No
Weighted Ensemble vs. EfficientNetV2-S	0.048835	Yes	+0.00139	0.00005	Yes
Soft Ensemble vs. EfficientNetV2-S	0.071394	No	+0.00152	0.00003	Yes
Swin Transformer-T vs. EfficientNetV2-S	1.000000	No	+0.00152	0.00146	Yes

**Table 12:** Performance comparison with representative recent studies on pediatric pneumonia detection from chest X-ray images.

Study	Dataset	Model	ACC (%)	Sensitivity (%)	Specificity (%)	AUC (%)	Key Characteristic
Liang & Zheng (2020) [20]	Pediatric Chest X-ray	Residual CNN + Dilated CNN	90.50	96.70	–	95.30	Early CNN-based transfer learning
Ayan et al. (2022) [21]	Pediatric Chest X-ray	ResNet50 + Xception + MobileNet Ensemble	95.83	97.76	92.73	95.21	CNN ensemble approach
AlGhamdi (2024) [24]	Pediatric Chest X-ray	MobileNetV3 (Transfer Learning)	95.82	98.00	97.00	–	Lightweight CNN architecture
Cahyani et al. (2024) [28]	Pediatric Chest X-ray	Vision Transformer (ViT)	97.78	97.88	–	–	Vision Transformer-based approach
Radočaj et al. (2025) [32]	Pediatric Chest X-ray	InceptionResNetV2 + Mish	97.61	97.61	–	–	Transfer learning with Mish activation
Chauhan et al. (2025) [25]	Pediatric Chest X-ray	LightPneumoNet	94.23	99.49	85.47	–	Lightweight CNN prioritizing sensitivity
Proposed (Soft Ensemble)	Pediatric Chest X-ray	EfficientNetV2-S + Swin Transformer-T	96.96	98.72	94.02	99.21	Best hold-out performance
Proposed (Hybrid CNN–Transformer Stacking)	Pediatric Chest X-ray	EfficientNetV2-S + Swin Transformer-T + Logistic Regression	97.37 *	97.96 *	95.77 *	99.67 *	Best cross-validation performance with the fewest false-negative predictions

Results marked with an asterisk (*) are from five-fold cross-validation.

## Data Availability

The dataset used in this study is publicly available through the Kaggle repository Pediatric Pneumonia Chest X-ray (Andrew MVD): https://www.kaggle.com/datasets/andrewmvd/pediatric-pneumonia-chest-xray (accessed on 1 February 2026). The code and additional materials used in this study are available from the corresponding author upon reasonable request.
